# An Internet of Things Sensor Array for Spatially and Temporally Resolved Indoor Climate Measurements

**DOI:** 10.3390/s22124377

**Published:** 2022-06-09

**Authors:** Alexander Rusch, Thomas Rösgen

**Affiliations:** Institute of Fluid Dynamics, ETH Zurich, 8092 Zurich, Switzerland; roesgen@ethz.ch

**Keywords:** COVID-19 pandemic, CO_2_ measurement, airborne transmission, indoor climate sensing, internet of things, wireless sensor array, ultra-wideband ranging

## Abstract

The COVID-19 pandemic has emphasized the need for infection risk analysis and assessment of ventilation systems in indoor environments based on air quality criteria. In this context, simulations and direct measurements of CO_2_ concentrations as a proxy for exhaled air can help to shed light on potential aerosol pathways. While the former typically lack accurate boundary conditions as well as spatially and temporally resolved validation data, currently existing measurement systems often probe rooms in non-ideal, single locations. Addressing both of these issues, a large and flexible wireless array of 50 embedded sensor units is presented that provides indoor climate metrics with configurable spatial and temporal resolutions at a sensor response time of 20 s. Augmented by an anchorless self-localization capability, three-dimensional air quality maps are reconstructed up to a mean 3D Euclidean error of 0.21 m. Driven by resolution, ease of use, and fault tolerance requirements, the system has proven itself in day-to-day use at ETH Zurich, where topologically differing auditoria (at-grade, sloped) were investigated under real occupancy conditions. The corresponding results indicate significant spatial and temporal variations in the indoor climate rendering large sensor arrays essential for accurate room assessments. Even in well-ventilated auditoria, cleanout time constants exceeded 30 min.

## 1. Introduction

The challenges posed by the COVID-19 pandemic have highlighted the need for further development of disease-spreading models, in this case, specifically for airborne virus transmission via aerosol transport.

Considering indoor scenarios, in particular, the employment of detailed computational fluid dynamics (CFD) simulations [1,2,3], simpler zonal [4,5,6], or even multizone simulations [7,8,9,10,11] appears feasible and may help to solve some of the specific ventilation issues. The capture of turbulent flow features at the full resolution scale, however, remains a limiting feature, and some degree of physical approximation is usually required [12]. This, in turn, generates a need for code calibration and adaptation to the scenario under investigation. For complex settings including forced ventilation, simulations and theoretical models show large discrepancies [4,13] such that experimental measurements present an attractive and necessary tool for validation.

Local CO_2_ concentrations are directly linked to human exhalation and serve as a marker for the transport of aerosols with a viral load [14,15]. Especially indoors, infection risks can be estimated from measurements of CO_2_ concentrations at inhalation height [16]. Numerous indoor air quality (IAQ) measurement systems already exist and typically use nondispersive infrared (NDIR) sensors. The presented work also utilizes that very sensor technology due to a trade-off between accuracy on the one hand and availability, as well as unit price, on the other hand. It must be noted, though, that recent advancements in gas sensor technology promise further improvements in terms of sensitivity and selectivity [17,18].

Most indoor climate sensing systems focus on “smart monitoring” [19,20] in the context of multi-room settings [20,21,22,23,24,25,26,27,28], e.g., they provide room-specific air quality warnings [29] via webserver/smartphone or ventilation control systems. These internet of things (IoT) devices not only enable the direct monitoring and control of IAQ on an individual smart home basis [30,31] but present a means of generating extensive cloud datasets that are analyzed to understand and optimize air flow paths and, ultimately, improve living [32] and working [33] conditions at a generalized scale. Such big data approaches, for example, utilize cloud processing to extract insights about air quality by applying machine learning techniques [32,33].

Existing indoor sensor networks are typically not aware of their mesh topology such that data are only visualized and inspected on a per-node basis, and manual mapping of sensor locations to the respective sampled datasets is necessary [26,27,28,29,34,35,36]. Furthermore, rooms are frequently only sampled in a single [21,24] or very few locations [22,35] instead of resolving entire rooms spatio-temporally with high resolution. Therefore, such systems are insufficient to capture the spatio-temporal complexity of natural and forced convective indoor flows. Furthermore, the respective measurement devices are often mounted in locations not representative of inhalation such as directly to a wall or ceiling. However, CO_2_ spreading in large rooms is inhomogeneous, inherently unsteady, and can feature significant stratification [16], which renders single-point measurements in non-ideal locations inadequate to assess infection risks [37]. Furthermore, the effectiveness of countermeasures and ventilation systems can only be evaluated from measurements that capture both spatial and temporal trends with high resolution.

To address these issues, in the scope of this work, a large and scalable 50-unit sensor array has been developed that provides spatially and temporally resolved room climate measurements in typical indoor environments, for example, seminar rooms and larger lecture halls with or without active ventilation. The system is designed for temporary deployment as a high-resolution measurement instrument rather than being permanently installed as part of smart building infrastructure. The battery-powered wireless measurement units can be flexibly distributed in a target room and placed at inhalation height. Besides the local CO_2_ concentration, temperature, relative humidity, and barometric pressure are measured. Despite being developed during the COVID-19 pandemic, the sensor array addresses the much wider topic of general indoor air quality measurement and assurance [20,21,22,23,24,25,26,27,29,34,38].

For large rooms and arbitrary sensor unit placement, reconstructing the 3D locations of all 50 sensor units manually is non-trivial, tedious, and error-prone. To bypass this problem, the sensor nodes are augmented with an anchorless ultra-wideband (UWB) ranging capability. Fused with an accurate height profile determination from calibrated barometric pressure readings, the sensor mesh topology is reconstructed with a mean Euclidean 3D position error of 0.21 m.

In the following, the design considerations, technical implementation, and measurement results for the 50-node IoT sensor array are described. The system was deployed during the COVID-19 pandemic to dynamically monitor the room climate and CO_2_ concentrations in various auditoria at ETH Zurich. Therefore, the presented results correspond to measurements under real occupancy conditions. The data indicate that, in particular, the time constants of the changes in the room climate have to be considered carefully.

The developed indoor sensor network is the first to use anchorless UWB ranging fused with barometric sensing for network topology awareness enabling the reconstruction of 3D indoor climate maps. Existing non-location-aware sensor networks visualize the data of the sensor nodes in the form of individual graphs. For large networks, this is impractical and does not allow for the easy detection of multi-scale spatio-temporal trends. In contrast, the proposed sensor network enables the creation of 3D evolution maps that provide intuitive insights into large datasets even for rooms with complicated topologies. Furthermore, a novel indirect approach for calibrating the CO_2_ sensors of all measurement nodes via a single reference unit is implemented. This work is further distinguished from most other indoor climate sensor networks by its size, the use of fully customized commercial sensing nodes, and the employed IoT approach that does not stream any data to the internet/into a third-party cloud but gathers and processes the information using only a local network and a fully (physically and virtually) operator-controlled server backend. To the best of the authors’ knowledge, no such large, flexible, robust, and location- as well as data privacy-aware IAQ sensing array has previously been reported.

## 2. Materials and Methods

The design and implementation of the sensor array were driven by a series of specific requirements, geared towards repeated application in different, possibly unknown indoor environments.

### 2.1. Resolution

The requirements on resolution include the spatial, temporal, and signal domains. It was considered sufficient to place sensing nodes at approximately 2 m distance for “high-resolution” measurements, comparable to typical values for social distancing. With a target size of the array of 50 nodes, this enables a regular coverage of around 200 m^2^.

Similarly, a temporal resolution of one sample per two minutes and node was considered useful. This figure relates to the need for also recording concentration changes during the room emptying phase where the effectiveness of the ventilation system is of particular interest.

Finally, the sensitivity of the individual nodes should be sufficient to detect—at least locally—the CO_2_ emissions of a single person. Assuming a production per person of approximately 0.7 g/min and a local “observation volume” of 10 m^3^, this translates into a value of 57 ppm/min. This figure has to be compared to the noise level and temporal stability of the actual CO_2_ sensors employed in the sensing nodes.

As will be detailed later, the sensing node functionality was extended to include a spatial ranging capability to automate the process of reconstructing the sensor node placement. Again, the resolution of the UWB ranging modules and the additional barometric height sensing was chosen to be better than 1 m for accurate position measurements.

### 2.2. Remote Operation

The second group of requirements was derived from the need to quickly place and operate the array in different environments with changing configurations.

This led to a design of the embedded nodes for decentralized, “headless” operation with a wireless transmission capability, receiving configuration/control information from and sending measurements to a remote server. Each node would have to include WiFi transmission capability, and a communication protocol compatible with the existing wireless network had to be chosen. The details of the resulting IoT constellation will be discussed further below. Importantly, the client–server architecture safeguards the array’s operation against the failure of individual measurement nodes.

Furthermore, the nodes’ power management should enable operation over extended periods of time (e.g., several days continuously) to monitor long-term trends. A large rechargeable battery was to be included in each node, and the hardware components had to be selected to facilitate a low-power sleep state between measurements. The sensor nodes were specifically designed as battery-powered devices since power adapters would have seriously downgraded remote operability and configuration flexibility, especially for the large number of measurement nodes involved.

In addition to these requirements that translate directly into hardware selection criteria, there were further design considerations that had an impact primarily on the software design. One was the scalability of the array, implying that the number of nodes to be operated should be easy to increase (or decrease) without any reprogramming required. Furthermore, provisions had to be included to recalibrate the sensing units remotely so as to maintain consistency of the readings across all sensors.

### 2.3. Hardware Architecture

The sensor array structure will be discussed considering two points of view: Section 2.3.1 describes the measurement system from a macro perspective including auxiliary systems while Section 2.3.2 goes into detail on the composition of the individual sensor units.

#### 2.3.1. IoT Mesh Structure

The measurement system consists of three functional blocks:

1. A flexibly configurable number of independently and asynchronously operating embedded measurement nodes. Currently, 50 units are available.
2. A reference measurement unit that provides calibration values for the measurement nodes. This is necessary as the installed CO_2_ sensors require recalibration at the beginning of each measurement campaign for best accuracy. Section 2.5.1 focuses on the CO_2_ sensor calibration procedure. The reference unit operates sensor hardware identical to the measurement nodes.
3. A server acting as a backend that provides remote management functionalities for the sensor array and logs the measurement data in a database (refer to Section 2.4.5). Furthermore, the server acts as the Message Queuing Telemetry Transport (MQTT) broker of the IoT mesh (refer to Section 2.4.1).

**Figure 1:**
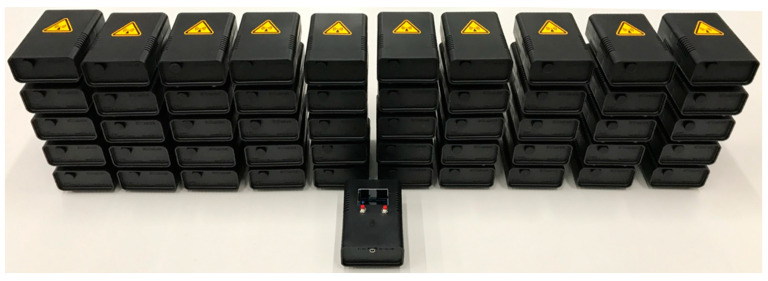
All 50 assembled sensor nodes with the reference unit in front.

Figure 1 shows the entire ensemble of 50 measurement nodes as well as the reference unit. Note that the reference unit is the only device with a display and buttons. A simplistic graphical user interface (GUI) implemented in the reference unit guides the operator through the calibration process (detailed in Section 2.5.1). The measurement nodes have no provisions for user interaction beyond a magnetically activated power switch.

#### 2.3.2. Measurement Node Design

The nodes are based on a modular design, using highly integrated commercial-off-the-shelf (COTS) components. A bus system using the Inter-Integrated Circuit (I^2^C) and Serial Peripheral Interface (SPI) protocols was chosen to connect the individual sensors, enabling fast assembly of the components without the need for dedicated interconnect circuitry. The bus architecture also allows for the later addition of further environmental sensing components such as particle size analyzers or volatile organic compound (VOC) detectors [19].

The key components integrated in a single node are, at present:

1. A master microcontroller with WiFi transceiver (Espressif Systems ESP32/WROOM32).
2. A CO_2_, temperature, and humidity sensor (Sensirion SCD30).
3. A high-precision barometric pressure sensor (Bosch BMP390).
4. A UWB ranging module (Decawave DWM3000).
5. A 6600 mAh Li-ion rechargeable battery pack.

**Figure 2:**
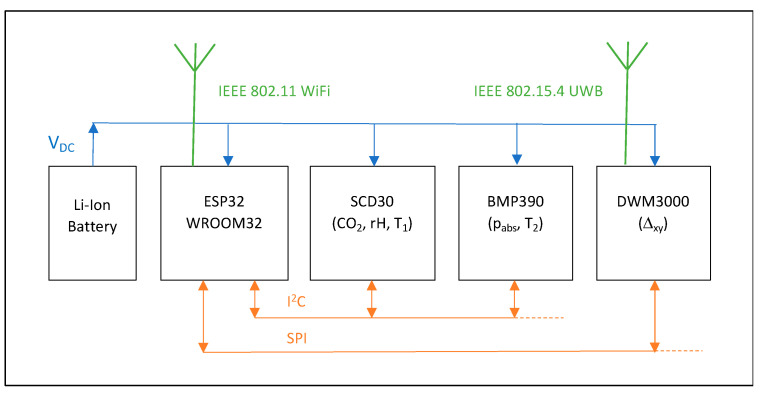
Block diagram of the node hardware.

A block diagram of these components is shown in Figure 2.

The specifications of the two main sensor components SCD30 and BMP390 are detailed in Table 1. The respective data are extracted from [39,40,41]. For further specifications, refer to these references.

All sensors are, in principle, capable of rapid data acquisition. In the interest of power conservation, though, the nodes operate currently at a (reconfigurable) sampling rate of one sample every two minutes. The data from each sampling sweep (CO_2_ concentration, relative humidity, barometric pressure, temperatures T_1_ and T_2_) are packaged into a message and transmitted to the data server via WiFi using the MQTT protocol (see Section 2.4.1).

The ranging module is activated and read out only at the beginning of a measurement cycle since the nodes’ position is assumed to remain unchanged during the subsequent measurements. This assumption is not valid during the pressure sensor calibration setup, though, where all nodes are temporarily moved to the same height level (refer to Section 2.5.2).

### 2.4. Software Implementation

The measurement nodes’ firmware was designed with special emphasis on uniformity such that all units run the same software differing only by a node-specific identifier (ID). At the same time, the software enables the independent operation of all nodes. This facilitates the flexible addition or removal of sensor nodes used in a measurement campaign and eases the future process of scaling up the array.

**Figure 3:**
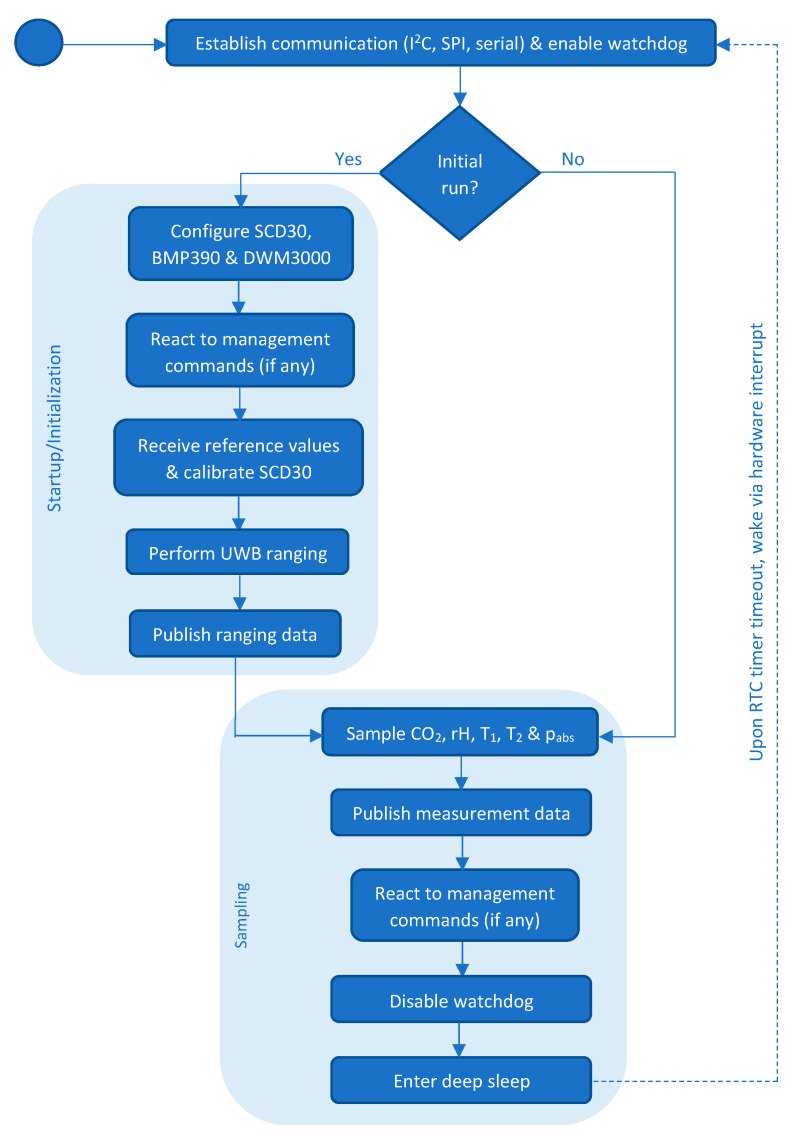
Program sequence of the measurement node software.

Figure 3 provides a simplified functional overview of the program structure of the measurement nodes. Details regarding the different functionalities of the software will be discussed in the subsequent sections.

#### 2.4.1. MQTT Structure

MQTT was chosen as the network protocol to transmit data from the measurement units to the server backend and to implement management functionalities for the sensor array. MQTT adheres to a strict client–server, publish–subscribe paradigm where all network traffic passes through the server, which is called the *broker* in MQTT terms. Messages are published under so-called *topics*, to which MQTT *clients* can subscribe. The broker routes incoming information to all clients that have subscribed to the corresponding topic under which a message is published. However, the broker itself remains passive and cannot publish any messages by itself. In the specific setup at hand, the measurement units, as well as the reference unit, act as MQTT clients, while the server backend acts both as the MQTT broker and client at the same time. As such, the server runs an MQTT broker software as well as a concurrent daemon that connects to the local host as an MQTT client, receives all nodes’ measurement data, and logs them in a database. Upon startup of the sensor array, the broker relays a retained MQTT message containing the reference unit’s calibration data to all sensor nodes as they come online. A multitude of MQTT message topics allows clients (including the server backend) to selectively gather and process:

1. Reference unit measurement data (“calibration values”).
2. Sensor unit ranging data.
3. Sensor unit measurement data.
4. User-triggered remote management commands.
5. Automatic client management commands.
6. Operational status messages.

#### 2.4.2. Startup/Initialization

As indicated in Figure 3, the sensor nodes operate in two distinct modes: Startup/initialization mode and sampling mode. During the startup of the sensor array, each node performs three tasks:

1. Configuring the SCD30, BMP390, and DWM3000 sensor modules.
2. Receiving the calibration values from the reference unit and calibrating the CO_2_ sensor accordingly (refer to Section 2.5.1).
3. Performing UWB-based ranging with all other nodes that can be reached and sending the ranging information to the database backend.

In addition, during initialization, the sensor nodes may receive management commands and act upon them. For instance, the operator can remotely instruct nodes in the IoT array to restart. 

#### 2.4.3. Failsafe Operation

When operating dozens of sensor units, scale-up-specific problems start to appear that are not encountered with a prototype-scale lower number of units. The erroneous behavior of single nodes must be accepted as inevitable and factored in. For example, single units may not be able to successfully range with their peers. Moreover, they may fail to return messages when trying to connect to the WiFi or the MQTT network. Furthermore, nodes may become stalled during operation due to irregular sensor behavior or hardware failures. With a measurement interval of two minutes per node, at full array capacity, the IoT mesh yields 50×60/2=1500 measurement cycles per hour—each cycle consists of multiple sensor readouts and network connection attempts. Therefore, it is crucial to ensure the failsafe operation of the single measurement nodes and—even more importantly—of the sensing array as a whole. Fault tolerance was implemented considering three aspects.

Due to the decentral and symmetric software design, a faulty sensor node does not influence the rest of the array. Except for the UWB ranging stage, during the startup of the IoT mesh, the sensor nodes operate asynchronously and independently from each other. No node synchronization is required once the sampling stage is entered. 

A deep sleep mode (refer to Section 2.4.4) powers down the host microcontroller and most peripherals between measurements. The wake-up from sleep mode effectively resets possibly erroneous internal states of the microcontroller. That is, even if a node is faulty, a possibility is created that it returns to a valid state after the next wake-up from a deep sleep. 

Finally, a timer-based hardware watchdog is present in each node that resets the sensor units in case they become stuck during operation (compare Figure 3). While awake, the sensor units must periodically reset their watchdog so as to indicate that the program sequence is actively progressing. Failure to do so causes the watchdog to time out and signals a potentially hung sensor unit. Upon timeout, the host microcontroller is reset, forcing the node into a clean startup.

#### 2.4.4. Sleep Mode and Power Consumption

The sensor nodes are battery-powered to ensure maximum configurational flexibility and ease of operation. This poses efficiency constraints on the IoT mesh to maximize the runtime of the nodes. For this purpose, the measurement units enter a low-power deep sleep mode of fixed duration between measurements. The sleep interval effectively defines the maximum frequency at which the nodes send data to the server and is currently set to two minutes. Once the measurement data are transmitted to the server backend, the host microcontroller powers down all components including its own subsystems that are not needed to recover the node from its deep sleep state. During sleep, the sensor node is unresponsive and cannot receive MQTT messages. Table 2 lists the measurement node components and subsystems along with their respective states during deep sleep.

Before entering deep sleep at the end of a measurement cycle, a timeout of the real-time clock (RTC) is registered as a wakeup source. This causes the RTC controller and the ultra-low-power (ULP) coprocessor to remain up and running as the ESP32 enters sleep mode. In this state, the ESP32 draws a current of only 0.2 mA. Upon timeout of the RTC timer, the ESP32 is woken via hardware interrupt from the ULP coprocessor and starts up normally just as it would during initial powering—with one crucial exception: Before going into deep sleep, a binary flag is set in the RTC slow memory indicating whether the sensor node has already passed its initialization stage including UWB ranging and CO_2_ sensor calibration. The ESP32 is then configured to keep the RTC slow memory powered even during sleep. At each wake-up, the initialization flag is checked so that ranging and calibration are only performed once. This avoids re-running these time- and power-consuming stages of the sensor array operation even though the microcontroller is newly initialized every time it is woken.

For additional power consumption optimization, the sensor node software is designed in such a way that, during the sampling stage, the uptime of the units in between sleeps is as short as possible. Typically, a sensor unit is awake for less than one second per measurement cycle. However, this is strongly dependent on the WiFi conditions, as establishing the network connection takes up the largest part of the uptime.

The DWM3000 module is only used during the UWB ranging in the initialization stage of the sensor unit (compare Figure 3). Afterward, it is permanently powered off and also remains inactive during deep sleep. The BMP390 sensor is triggered by the ESP32 to perform a single measurement “on demand”. Therefore, it is inactive by default and only wakes once per measurement cycle to acquire a single sample. The SCD30, on the other hand, needs multiple samples for its CO_2_ readings to converge. As such, the response time constant is inversely proportional to the SCD30’s sampling frequency. Due to this accumulating measurement principle, the SCD30 cannot be triggered to perform a single instantaneous measurement. Instead, the sensor samples continuously with the configured frequency and needs to be polled by the host for a new, converged reading. This has several implications for the measurement nodes:

1. The SCD30 sensor cannot be powered down during deep sleep. It must remain up and running so that it can sample the necessary data points to provide a converged CO_2_ reading at the next measurement cycle of the node.
2. To avoid having the host stay awake for longer than necessary due to unsuccessful attempts at polling the SCD30, the sampling frequency of the sensor needs to be chosen fast enough such that a converged CO_2_ value is available to be read by the next time the ESP32 wakes up.
3. At the same time, the sampling frequency of the SCD30 should be set as low as possible such that the power-consuming sampling process of the SCD30 is not performed unnecessarily often.

The SCD30 CO_2_ sensor is the most power-consuming component of the sensor nodes next to the ESP32 processor since its operation includes driving an infrared light-emitting diode (LED) as an optical sampling beam. The manufacturer specifies the maximum current drawn by the sensor to be 75 mA [40]. As elaborated above, the sensor must sample periodically and cannot be powered down in sleep mode. For an optimum tradeoff between measurement accuracy and power consumption, the sampling frequency of the SCD30 and the sleep interval of the sensor units need to be adjusted. A reference sheet for the CO_2_ sensor relates sampling frequencies and response times [42]. This technical specification also prescribes a lower and upper bound on the sampling interval of [5 s; 60 s]. Ignoring this operating limit would result in inaccurate CO_2_ measurements. Given a desired temporal resolution of the sensor array of two minutes, an optimized SCD30 sampling interval of 20 s is selected.

Another power-intensive task is the UWB-based ranging. However, it is only performed once at the startup of the sensor array. Thus, its influence on the sensor unit’s total runtime remains limited—it consumes a small, fixed amount of the battery capacity available at the sampling stage.

While the effective capacity of the used rechargeable battery packs varies, the sensor units achieve an average continuous runtime of more than seven days with the chosen power management. This operating time easily covers typical time scales observed in indoor climate investigations. If necessary, the runtime of the sensor mesh can be prolonged by extending the sleep interval or shortened to increase the effective sampling rate.

#### 2.4.5. Database Structure and Management

In addition to the MQTT broker software and the daemon for receiving and logging measurement data, the backend server also runs a MariaDB database. It is structured into three separate tables storing information regarding:

1. Calibration/reference values.
2. UWB-based ranging.
3. Measurement data.

The calibration/reference table may contain as little as one entry if the calibration procedure via the reference unit is performed only once and in case it yields meaningful values right away. Typically, the reference measurement is repeated several times to ensure consistent values.

Each entry in the ranging table records the point-to-point distance between two measurement nodes as initiated by one of the two units. It is complemented by an entry of the same node-pairing but with reversed initiator. This table is populated upon startup of the sensor array and remains unchanged throughout the actual climate measurements. At most, this table contains n×(n−1) entries, where n is the number of sensor nodes deployed.

Whenever a measurement unit sends sampled data to the backend via MQTT, the logger daemon receives it and creates a new entry in the measurement data table. Each entry in this table contains the unique ID of the measurement unit and the sampling timestamp. Furthermore, the sampled entities, i.e., CO_2_ concentration, temperature measurement of the SCD30, relative humidity, temperature measurement of the BMP390, and barometric pressure, are logged. For a representative measurement campaign of 24 h with all 50 sensor nodes and the default two-minute sampling interval, this table contains up to 24×60/2×50=36,000 entries.

### 2.5. Sensor Calibration

Even though both the SCD30 and the BMP390 are factory pre-calibrated, recalibration for each measurement campaign is necessary for best accuracy. Section 2.5.1 describes how the CO_2_ sensor’s internal reference value is reset in each measurement node without having to manually expose each unit to a reference atmosphere. The alignment of the barometric pressure readings of the BMP390 sensors is similarly described in Section 2.5.2.

#### 2.5.1. CO_2_ Calibration

The Sensirion SCD30 CO_2_ sensors must be calibrated before each measurement campaign. For that purpose, a reference measurement with a known CO_2_ concentration is needed. To avoid the large operational overhead of calibrating every individual sensor node in an environment of a known CO_2_ concentration, an indirect, two-stage calibration procedure is employed that makes use of a single reference unit. In doing so, a common sampling baseline is established that guarantees consistent sensor readings across the entire sensor network at the beginning of a measurement campaign.

First, the reference unit is calibrated—it contains the same SCD30 sensor as the measurement nodes. For this “self-calibration”, the reference unit is placed outdoors in fresh air and a two-minute CO_2_ measurement is triggered, sampling a known CO_2_ concentration of approximately 400 ppm [43]. At the end of the self-calibration measurement, the reference unit resets its own SCD30-internal reference such that the current sensor output corresponds to a CO_2_ concentration of 400 ppm. Next, the now-calibrated reference unit is used to perform a two-minute measurement at the target measurement site where the IoT mesh is to be deployed (“reference measurement”). Ideally, this is performed while the target location is still unoccupied such that the CO_2_ concentration in the entire room can be assumed to be homogeneous. In doing so, the reference/starting CO_2_ concentration at the measurement site is determined. Both the self-calibration and the reference measurement duration are chosen to conform with the sampling interval of the sensor array. Furthermore, the SCD30 sampling frequency of the reference unit matches the measurement units’ configuration. At the end of the reference measurement, the reference values are sent to the MQTT broker as a “retained message”, which all measurement units receive upon initial startup (refer to Section 2.4.2). Once the measurement nodes receive the reference values from the MQTT broker, they reset their own SCD30-internal CO_2_ reading to the reference value. This concludes the relay-style two-stage calibration, and the measurement nodes are ready to sample.

#### 2.5.2. Pressure Calibration

**Figure 4:**
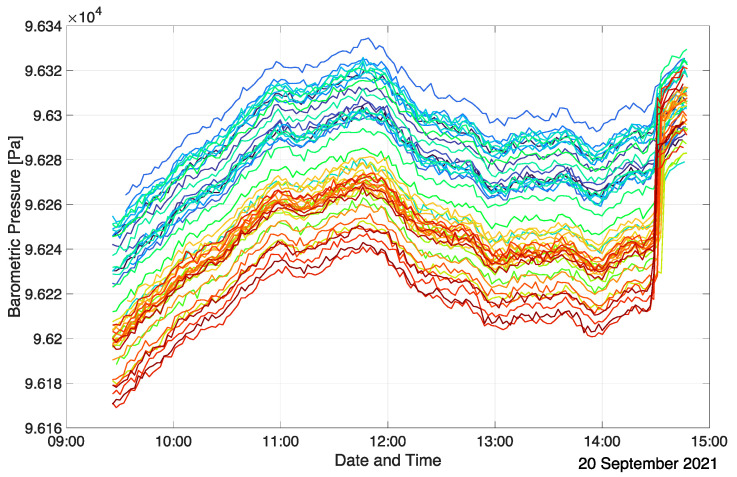
Uncompensated BMP390 values (prior to calibration) in an inclined auditorium.

**Figure 5:**
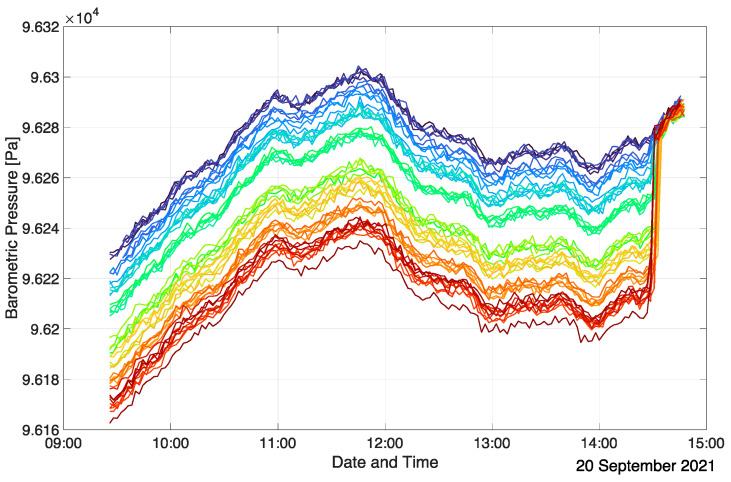
Compensated BMP390 values (after calibration) in an inclined auditorium.

The BMP390 senses absolute barometric pressure, which is used to determine the vertical position of each measurement node with respect to other units. As such, differences in the barometric pressure readings are of interest to determine relative height profiles. Even though the BMP390 sensors are factory calibrated, they exhibit a spread in absolute barometric pressure readings in the order of +/−0.5 hPa, corresponding to height differences of approximately +/−4 m [41]. To enable precision height measurement comparisons between the sensor units, a common reference needs to be established. For this, all sensor units are positioned at the same elevation level, and a few barometric readings are performed. Typically, the floor of the investigated room is chosen as the calibration level since it is readily referenced during post-processing. Any one of the units’ barometric pressure values at the calibration level can be chosen as a reference, and the offset of each BMP390 reading to this reference is then used to compensate for the sensor-specific variations. In a typical measurement campaign, the barometric pressure calibration is performed at the end of the actual measurement before the deactivation of the array. Once calibrated, the BMP390 readings are highly consistent even over long measurement periods. Figure 4 shows the BMP390 sensor readings of all sensor units of an exemplary measurement run (inclined auditorium case, see Section 3.2) in an uncompensated form, whereas Figure 5 depicts them after calibration. Each graph and color represent a single measurement node. In this specific setup, the sensor units were located at nine discrete heights during the measurement, which is reflected in the compensated barometric pressure readings as nine time-varying ensembles of barometric pressure values. The calibration procedure is evident from the sudden change in the barometric readings at the end of the measurement. In this case, the actual spread of the uncompensated barometric pressure readings is approximately half as large as stated in the BMP390 datasheet (+/−25 Pa corresponding to +/−2 m height difference). Calibration practically eliminates any relevant spread, and the relative accuracy of the sensors of +/−3 Pa (+/−0.25 m) is exploited. The changes in the barometric pressure over the course of the measurement are solely due to atmospheric fluctuations. This is evident from the highly correlated evolution of the different BMP390 readings at every time step. Figure 5 indicates that the offset between grouped barometric pressure plots is almost perfectly constant over time. Thus, the relative vertical positions of the sensor units are reliably determined.

Due to the high precision of the pressure readings, once calibrated, these values are more accurate than the UWB lateral ranging values and thus stabilize the spatial reconstruction algorithm and reduce the number of positional ambiguities.

## 3. Results and Discussion

As described above, the measurement of local CO_2_ concentrations is the key purpose of the sensor array as the production and exhalation of the gas by humans is directly linked to the aerosolized virus load produced. Differences in the diffusivity between CO_2_ and small aerosol droplets are sufficiently small for flows dominated by natural convection.

The following sections discuss the results from a measurement campaign in a large at-grade seminar room (Section 3.1) and a campaign in an inclined lecture hall (Section 3.2). Additionally, a discussion on the ventilation time constants is provided in Section 3.3. Furthermore, the accuracy of the proposed self-localization strategy via combined UWB ranging and barometric sensing is assessed in Section 3.4.

Both in-field measurement campaigns (Section 3.1 and Section 3.2) were conducted over several hours under real occupancy conditions and include distinct events such as people entering and leaving the rooms. Evolutionary 3D maps of the complete datasets of both campaigns are available as Videos S1 and S2 in the Supplementary Materials. Accompanying annotations embedded in the videos highlight significant events. It is highly recommended to inspect these animations in addition to the static plots provided in this section.

### 3.1. Measurements in an At-Grade Seminar Room

**Figure 6:**
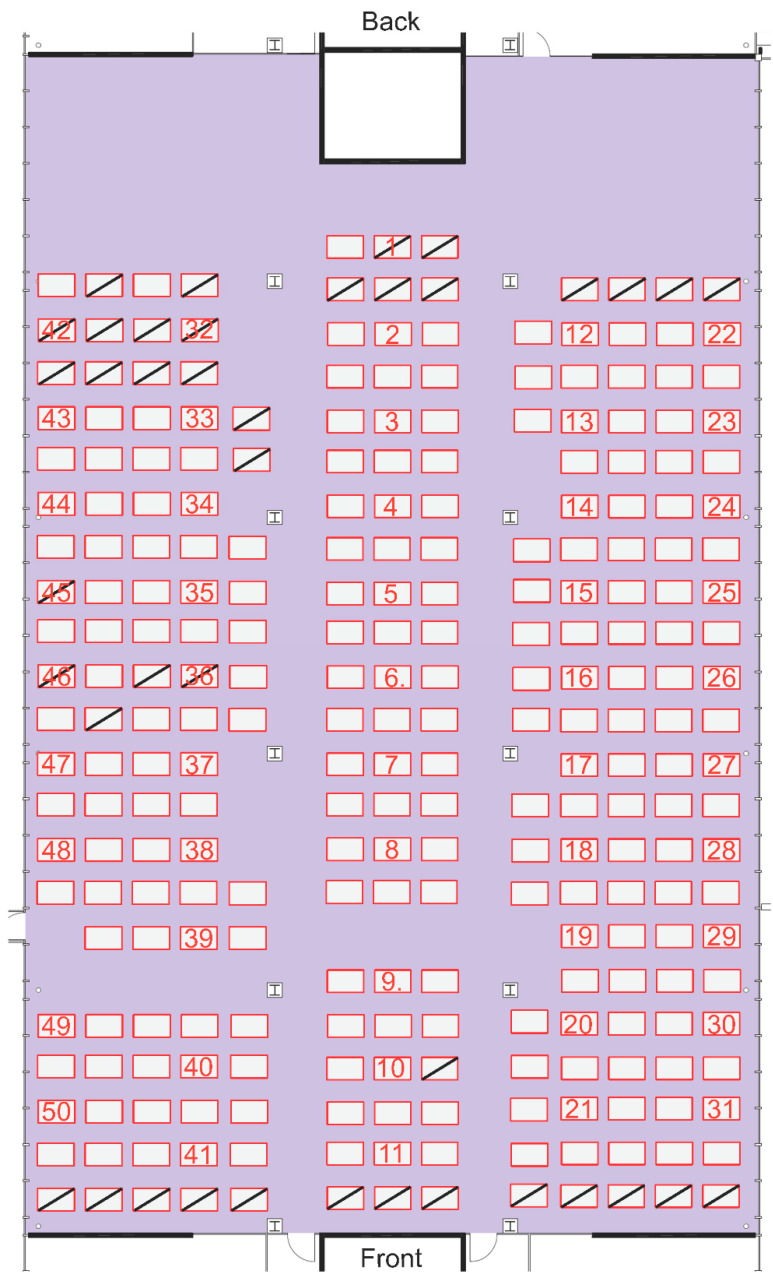
Layout of at-grade seminar room with measurement unit placements annotated. Unoccupied desks are crossed out.

The first measurement campaign was conducted on 27 August 2021 from 2:30 p.m. to 6:45 p.m. during an exam at ETH Zurich. The investigated room has a size of 1400 m^2^, in which 226 students were seated at individual and separate desks. This particular room is normally used for technical drawing classes for architectural and civil engineering students and also serves as an exam room due to its large size and the possibility to seat students at individual desks. The measurement started while the room was still unoccupied such that the transients from students entering could be captured and compared against the baseline. The room features two doors in the front and one door in the back, which were kept open during the entire duration of the exam/measurements. The sensor units were evenly distributed in the room and placed directly on the desks. Figure 6 shows the schematic topology of the examination room with the location of the 50 sensor units annotated by their respective IDs. Note that unoccupied desks are marked by diagonal lines.

**Figure 7:**
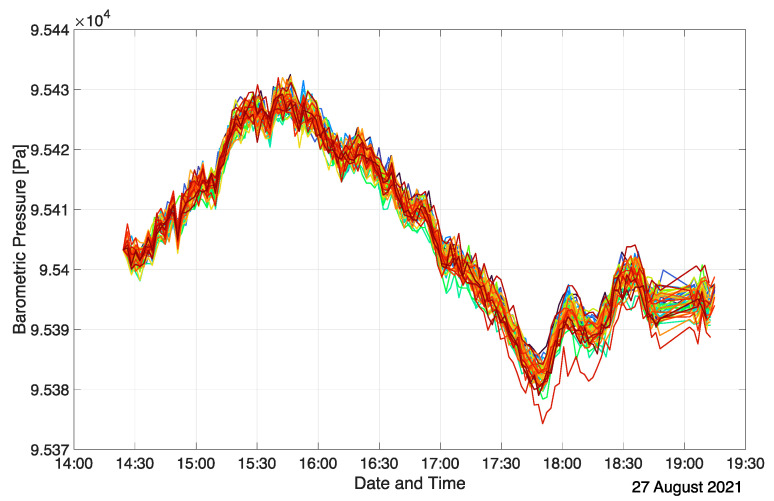
Calibrated barometric pressure readings in at-grade exam room. Sensor unit IDs are color-coded.

Since the entire room is at-grade, so were the measurement units. As a result, barometric pressure readings must only vary due to atmospheric pressure fluctuations and coincide after calibration, as can be seen in Figure 7. Note that the barometric pressure sensor calibration was performed prior to the measurement. The slightly diverging plots towards the end of the measurement indicate that some BMP390 sensors drifted over the course of the measurement campaign. However, the drift is minor with the largest absolute discrepancy of 10 Pa. Furthermore, in this case, the inconsistency is irrelevant for the room topology reconstruction as all sensor units were placed at the same height.

**Figure 8:**
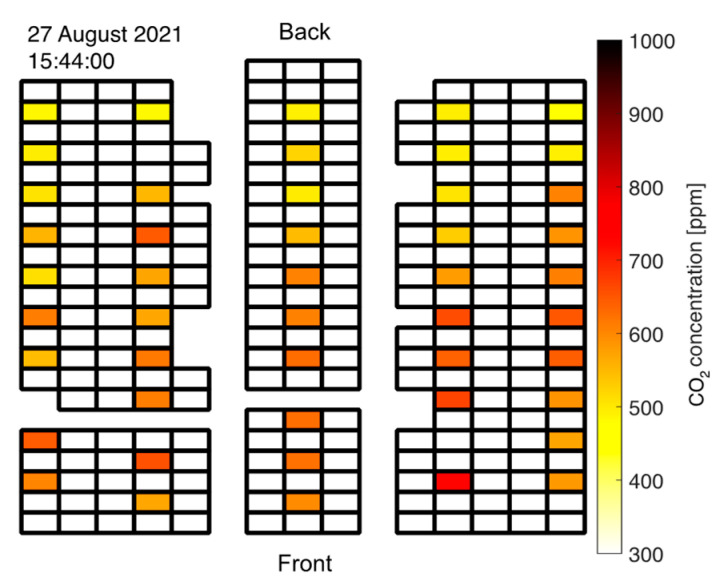
CO_2_ concentration gradient from back to front during seating of students prior to exam in at-grade room.

When students started entering the room through the back door, the CO_2_ readings increased rapidly with a distinct gradient from back to front as visualized in Figure 8. This conforms with the applied seating strategy: Students were seated in the front first and rows in the back were occupied later. Note that this highly unsteady behavior is best viewed in the Video S1 supplied in the Supplementary Materials.

**Figure 9:**
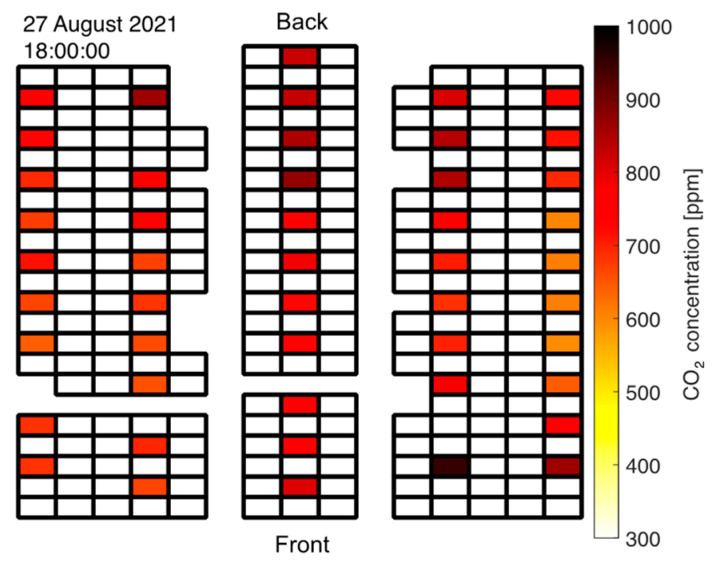
CO_2_ concentration gradient from front to back as students left the at-grade room after the exam.

A similar, yet spatially opposite effect was observed when the students left after the exam. In contrast to the orderly seating procedure prior to the exam, all students were allowed to exit the room at the same time through the back door after the exam. Naturally, the single back door presented a bottleneck such that the occupation of the room was shifted towards the back as students gathered in front of the exit. Accordingly, the zones of highest CO_2_ concentration first shifted towards the back of the room (see Figure 9) with the gradients pointing from front to the rear middle before the levels slowly dropped towards the baseline values when the room was completely empty again.

Again, this effect is best seen in the Video S1, Supplementary Materials. Note that the same effects during entering and emptying of the room are visible in the relative humidity readings. A zone in the front of the room featured high CO_2_ concentrations even after students had already cleared this area. This was caused by the supervisors gathering in that particular region after the exam. Figure 9 clearly depicts this in the lower right part of the plot corresponding to the measurement units 11, 21, and 31 (see Figure 6 for measurement unit mapping).

The local room temperature is an obvious parameter in the description of a room climate environment. Any observed non-uniformities have a direct impact on the natural convective and forced ventilation flows established.

**Figure 10:**
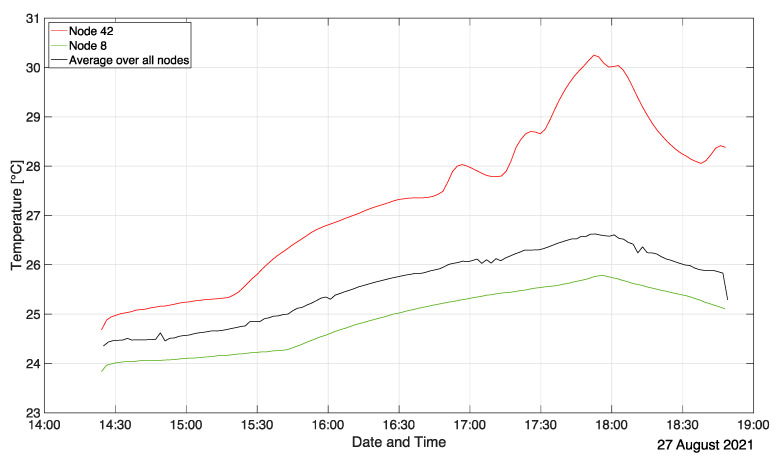
Temperature evolution in the large planar seminar room.

Figure 10 shows the temporal evolution of the temperature in the at-grade seminar room for a location close to a sun-lit window (node 42), in a shaded area (node 8), and the room average computed over all sensor nodes. Note that the average of the sensor readings is obtained at desk height and is not the same as, say, the average ceiling temperature. These visible differences highlight the need to include realistic thermal boundary conditions in any “high fidelity” numerical flow simulation. A constant increase in room temperature is visible in Figure 10 on average, as well as for the exemplified locations of nodes 8 and 42 right from the beginning of the measurement due to the room heating up from sunlight. The increased slope in the average profile and in the profile of node 8 around 3:45 p.m. marks the filling of the room. Note that node 42 already features a kink and a much steeper increase in temperature before students enter. This is due to direct sunlight impinging on the node and is evident for the entire ensemble of nodes 42–50 as they were all located at the sun-lit window front. This temperature inhomogeneity is best visualized in the Supplementary Video S1. Except for the temperature, at that point in time, the room climate was still relatively homogeneous. The corresponding local decrease in relative humidity is also apparent for nodes 42–50 (see Supplementary Video S1). Note that Figure 10 even allows one to infer shaded and sunny phases of the day during the exam depicted by the fluctuating temperature evolution of node 42 between 4:50 p.m. and 5:50 p.m. At around 5:50 p.m., students started leaving the examination room. Accordingly, the average room temperature, as well as the temperatures at nodes 8 and 42, decrease.

### 3.2. Measurements in an Inclined Auditorium

**Figure 11:**
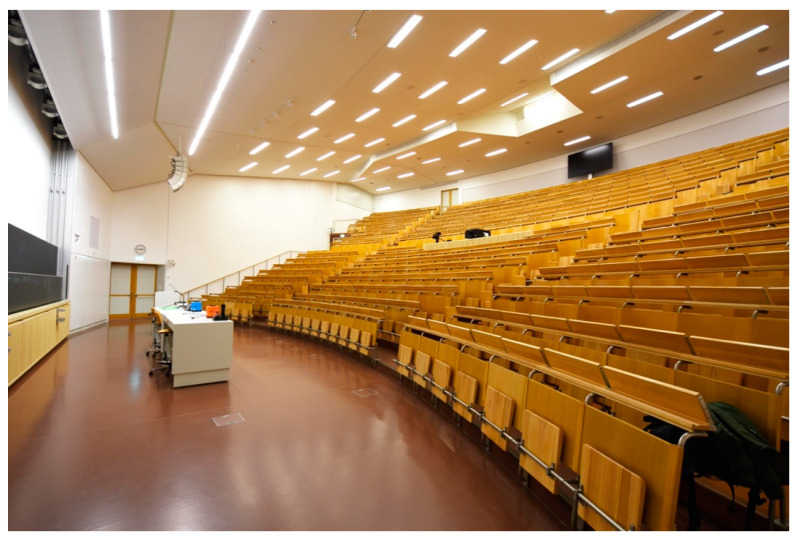
Inclined auditorium at ETH Zurich. Image taken from a front corner. Image used with permission from ETH Zurich.

The second room investigated with the sensor network is a large, inclined auditorium at ETH Zurich with a base area of 460 m^2^ and 600 seats. An image of the room as seen from a front corner is shown in Figure 11. The top seats in the back are located approximately 7 m above the front rows.

The measurement was performed on 20 September 2021 between 9:25 a.m. and 2:50 p.m. During that time, two subsequent introductory student events were carried out, both of which used the auditorium at approximately 50% capacity. No detailed seating plan as provided in Section 3.1 is available since students were allowed to join the events later, leave early, and move around/change seats. Again, the measurement was started prior to the first students entering to establish some reference data points in undisturbed room conditions. With an even distribution of the sensor units, every second seating row and approximately every fifth to sixth seat was covered by the measurement system. In this campaign, two features are of specific interest: First, the sloped topology of the room in combination with a ventilation system injecting air at the floor level and extracting at the ceiling should establish forced and natural convective flows directed towards the upper rear seating rows. Second, the capture of two subsequent events in the same room should provide insights into the cleanout and ramp-up time constants. The full 3D evolution map of all sensor readings is visualized in Video S2 available in the Supplementary Materials. Refer to this animation for the most intuitive presentation of the collected data.

**Figure 12:**
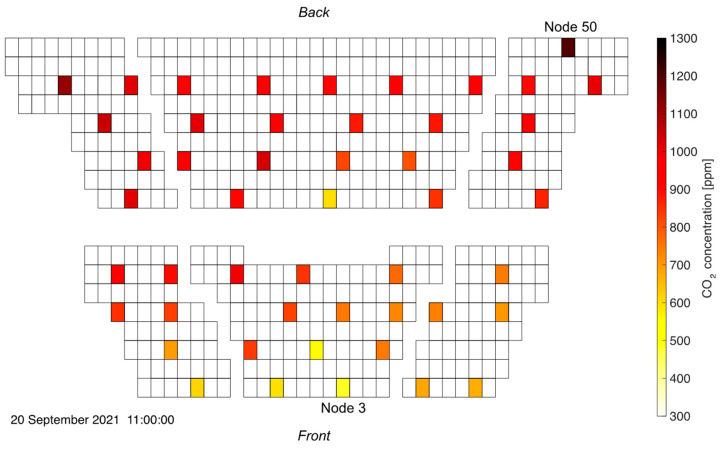
CO_2_ distribution in the inclined auditorium. A strong concentration gradient from front to back is visible.

Figure 12 shows the CO_2_ concentration distribution at 11 a.m. in a bird’s eye view. Each rectangle represents a seat in the lecture hall and the colored spots mark the locations of the measurement units. That particular time instance is chosen as it represents an extreme situation with close to maximum room average and top-level CO_2_ concentrations, while the sensor readings were not yet influenced by people leaving the room. One clearly recognizes the significant concentration gradient from front to back, with critical levels above 1200 ppm being approached in the rear. As expected, exhaled air is convected up the topological ramp of the inclined auditorium towards the rear. Clearly, seats in the front of the lecture hall are much less exposed to potentially virus-loaded air than seats further in the back. As was the case in the previous measurement campaign, relative humidity readings closely follow the CO_2_ concentration evolution. Refer to Video S2 in the Supplementary Materials for a side-to-side animation of both readings.

**Figure 13:**
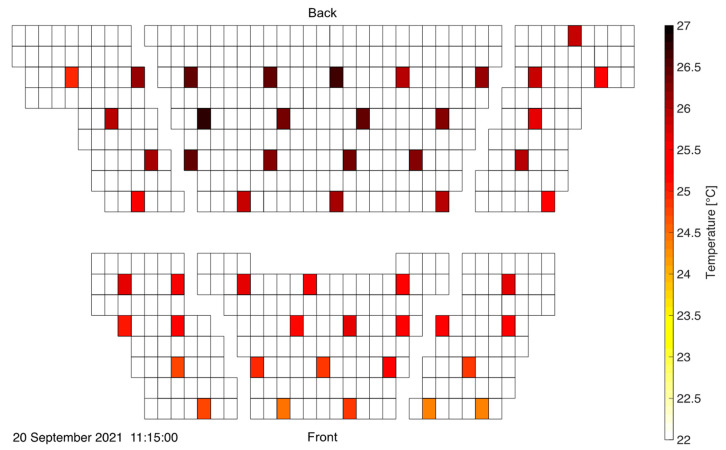
Temperature distribution in the inclined auditorium at the end of the first student event. Highest temperatures are observed in the rear of the room.

The significant contribution of natural convection to the overall upwards directed flow profile becomes apparent when inspecting the temperature distribution in the auditorium. Figure 13 depicts the temperature profile in the auditorium at the end of the first student event. Clearly, the rear middle region of the lecture hall exhibits the largest temperatures indicating that hot air is predominantly flowing and accumulating towards the back of the auditorium.

**Figure 14:**
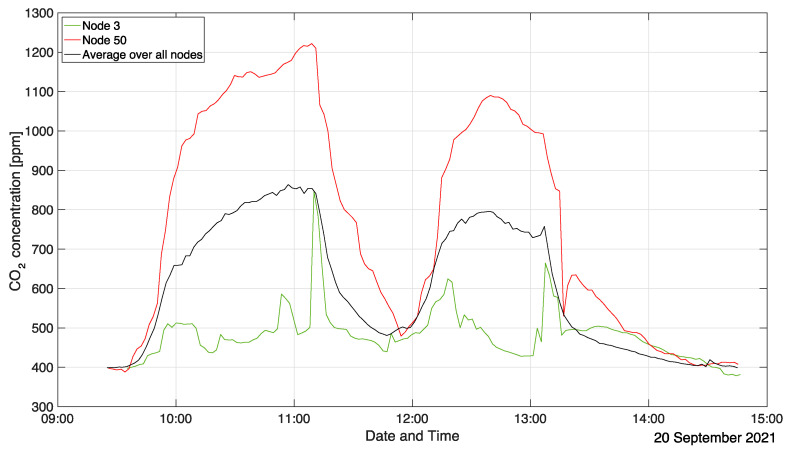
Local and average CO_2_ concentrations in the inclined auditorium.

When studying the ventilation in such an environment, a clear distinction has to be made between the average CO_2_ concentration (or temperature) and its local values. This is visualized in Figure 14 where the temporal evolution of the average value (across all sensor nodes) is compared with readings in a “hot spot” location in the rear (node 50) and a “clean” patch (node 3) at the front. See Figure 12 for the locations of nodes 3 and 50.

In this highly inhomogeneous environment, resolved measurements can provide the necessary values for tuning corresponding numerical simulations, which would normally rely on empirical turbulence and mixing models [12].

Figure 14 intuitively depicts the timeline of events during that measurement campaign: Between 9:40 a.m. and 10 a.m. students entered the room. Consequently, in that time frame, the average and local CO_2_ concentrations increased steeply. Around 10 a.m., the slope of the curves decreases, indicating the start of the first student event in the auditorium. The average CO_2_ concentration keeps increasing up until around 11 a.m., which was the time at which the first student event ended. Within the next 10 min, people exited the lecture hall. During that time, the average CO_2_ concentration remains constant, while it even keeps increasing at the location of node 50. At 11:10 a.m., all students had left the room marking the beginning of the clean-out phase. Students started entering the lecture hall again at 11:50 a.m. at a residual average CO_2_ concentration of 500 ppm for the second event. Even though fresh air conditions could not be fully recovered, due to the asymptotic behavior of the curve, a clean-out time of at least one hour can be estimated. The analysis of the second introductory student event starting at 12:15 p.m. follows the same reasoning except for the fact that both the CO_2_ concentration at node 50 as well as the room average show a distinct maximum around 12:40 p.m. that is not visible in the trendline of the first event. Furthermore, the maximum CO_2_ levels are lower than during the preceding event. This is because the room ventilation was ramped up during the second event at 12:40 p.m. to increase the air exchange in the lecture hall. Consequently, the clean-out phase after the second student event starting at 1:15 p.m. is also shorter. This time, the room returned to fresh air levels, which took approximately 70 min after the lecture hall had been emptied.

At this point, a remark has to be made on the array’s sensitivity. The majority of optical CO_2_ sensors are specified with sensitivities/noise floors of approximately 50 ppm CO_2_. While this appears to be sufficient to detect the emissions of a single person, such a measurement depends critically on the environmental conditions. In a large room with forced ventilation, the gas exchange rates of several thousand m^3^/h can easily be so large that the contributions of a single person fall well below that threshold. This remains a challenge if the array were to be used for measurements of realistic “point spread functions”, i.e., with local CO_2_ production rates on the order of grams per minute.

### 3.3. Ventilation Time Constants

**Figure 15:**
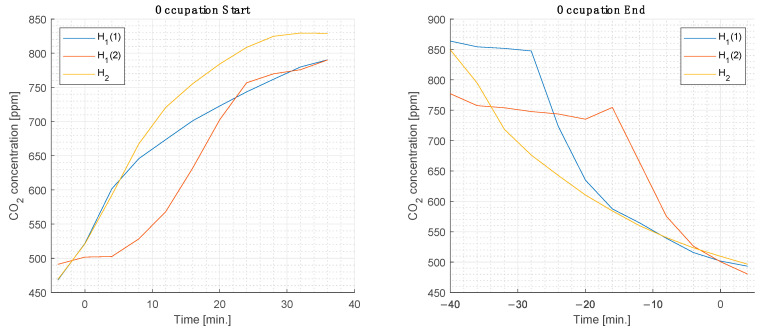
Rise and decay of CO_2_ concentration during room population and emptying phases.

A figure of specific interest is the decay rate of the CO_2_ concentration once a room has been emptied. This has obvious consequences for the overall occupation policy and turns out to also be one of the more difficult/decisive parameters to reproduce in any high-fidelity simulation. Here the readings of average CO_2_ concentrations in the two rooms presented before (H_1_: 460 m^2^, inclined; H_2_: 1400 m^2^, at-grade) are examined, both at the start of occupancy and during the cleanout phase after the audience had left (Figure 15).

One recognizes rather significant time constants in the order of several tens of minutes for both transient phases. It is noteworthy that even in the same room (H_1_), the time constants differ significantly. In the case of H_1_(2), as elaborated above, feedback control of the ventilation system was activated, in contrast to cases H_1_(1) and H_2_, where constant ventilation was maintained. The ramping up of the ventilation in the former case led to a reduction of approximately a factor of two in the ventilation turnover times.

### 3.4. Sensor Node Position Reconstruction

In order to demonstrate the functionality and accuracy of the ranging approach, a subset of ten sensing nodes was placed in a controlled environment with placement heights and separations determined by independent laser ranging. This allows one to compare the ranging results based on the UWB and barometric pressure data with the “ground truth”. Processing of the ranging data is performed with multidimensional scaling (MDS), specifically the SMACOF algorithm (**S**caling by **Ma**jorizing a **Co**mplicated **F**unction, [44]). Additionally, the algorithm is augmented to include the barometric node heights as constraints. These constraints prove essential for the MDS reconstruction in the absence of anchor nodes with known spatial reference positions.

**Figure 16:**
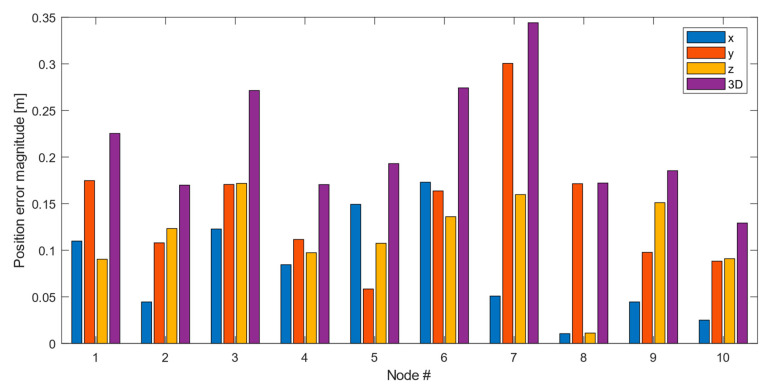
Position errors following MDS/SMACOF reconstruction.

Figure 16 displays the position errors with respect to the known ground truth positions. The error magnitudes are roughly the same in all three directions, and the mean Euclidean 3D error is 0.21 m. This value must be compared to the size of the sensing volume covered by the nodes, which was 4 × 5 × 2 m^3^.

Tests in larger rooms and locations have shown that ranging up to distances around 20 m does not pose a problem for the current implementation with the DWM3000 UWB modules. Nevertheless, it cannot be excluded that more complicated settings (e.g., array patterns without line-of-sight visibility of the nodes and cluttered environments in general) may pose some as of yet unknown challenges (loss of signal, multipath problems, etc.).

## 4. Conclusions

The paper presents a large IoT sensor array to monitor the variations in the indoor climate of large auditoria with high spatial and temporal resolution. While the development of the sensor array was driven by the need for rapid verification of numerical predictions in the management of such rooms under epidemic restrictions, the demonstrated functionalities point to a broader field of applications in the general area of indoor air quality assessment and control. Highly unsteady events, as well as significant spatial gradients in CO_2_ concentration, temperature, and relative humidity, are successfully captured and resolved, as well as visualized readily in 3D evolution maps. Based on scalable IoT and MQTT technology, the sensing array is extensible to even larger sizes. Through the use of highly integrated COTS components, the cost per node is kept low such that the hardware investment does not appear to be a limiting factor in this regard.

To overcome operational and management issues imposed by the size of the sensor network, a simple relay-style calibration method was developed that reduces the operator’s manual overhead to a minimum. Furthermore, a novel 3D sensor node positional awareness mechanism is presented that eliminates the need for spatial anchors by combining UWB ranging and barometric pressure sensing. Finally, special care was taken at the sensor array conceptualization stage to design the entire system and its software for a maximum level of robustness and data privacy-awareness.

The insights gained from the proposed sensor network enable identifying seating positions with high infection risks in lecture halls and optimizing temporal and spatial room occupation schedules such that they conform with cleanout times and local flow patterns. Furthermore, the large and high-resolution datasets can serve both as input for numerical simulations and their subsequent validation at different scales and levels of complexity.

Future work will focus on the incorporation of additional sensing components such as particle counters, as well as other gas (VOC) detectors to yield an even more complete air quality assessment. Furthermore, the extension of the array to a larger number of sensor nodes is anticipated. Moreover, as more and more datasets are collected, machine learning techniques may be used to infer IAQ metrics in rooms with, as of yet, untested topologies.

## Figures and Tables

**Table 1:** Sensor specifications.

Component	Measurement Range	Accuracy	Repeatability	Response Time (τ_63%_)	Temperature Stability
SCD30—CO_2_	400–10,000 ppm	+/−(30 ppm + 3% M.V. ^1^)	+/−10 ppm	20 s	+/−2.5 ppm/°C
SCD30—rH	0–100%	+/−3%	+/−0.1%	8 s	N.A. ^2^
SCD30—T_1_	−40–70 °C	+/−(0.4 °C + 0.023 °C * (M.V. ^1^—25 °C))	+/−0.1 °C	10 s	N.A. ^2^
BMP390—p_abs_	300–1250 hPa	+/−50 Pa (abs.); +/−3 Pa (rel.)	N.A. ^2^	N.A. ^2^	+/−0.6 Pa/°C
BMP390—T_2_	−40–85 °C	+/−0.5 °C	N.A. ^2^	N.A. ^2^	N.A. ^2^

^1^ Measured value. ^2^ Not applicable/not available.

**Table 2:** State of sensor node components during sleep mode.

Component	Subsystem	State During Deep Sleep
	CPU	Down
	Memory (ROM, SRAM, flash)	Down
	ULP coprocessor	Up
ESP32	WiFi, Bluetooth & radio transmitter	Down
	RTC controller & peripherals	Up
	RTC fast memory	Down
	RTC slow memory	Up
	Microcontroller peripherals	Down
DWM3000		Down
BMP390		Down
SCD30		Up

## Data Availability

The data presented in this study are available on request from the corresponding author. The data are not publicly available due to privacy reasons since the measurements were conducted in the presence of students and staff. The full measurement data are visualized in the Supplementary Videos.

## Supplementary Materials

The following supporting information can be downloaded at: https://www.mdpi.com/article/10.3390/s22124377/s1, Video S1. SuppInfo_evolution_map_atgrade_auditorium.mp4: Evolution of indoor climate in at-grade auditorium during exam, Video S2. SuppInfo_evolution_map_inclined_auditorium.mp4: Evolution of indoor climate in inclined auditorium during two subsequent student events
